# Hybrid coordination-network-engineering for bridging cascaded channels to activate long persistent phosphorescence in the second biological window

**DOI:** 10.1038/srep20275

**Published:** 2016-02-04

**Authors:** Xixi Qin, Yang Li, Ruili Zhang, Jinjun Ren, Mindaugas Gecevicius, Yiling Wu, Kaniyarakkal Sharafudeen, Guoping Dong, Shifeng Zhou, Zhijun Ma, Jianrong Qiu

**Affiliations:** 1 State Key Laboratory of Luminescent Materials and Devices, Guangdong Provincial Key Laboratory of Fiber Laser Materials and Applied Techniques, South China University of Technology, Guangzhou 510640, China; 2 School of Chemistry and Chemical Engineering, South China University of Technology, Guangzhou 510640, China; 3 Shanghai institute of optics and fine mechanics, Chinese Academy of Sciences 201800, China; 4 Escola de Engenharia de Sao Carlos, Universidade de Sao Paulo, 13566-590, Sao Carlos, SP, Brazil

## Abstract

We present a novel “Top-down” strategy to design the long phosphorescent phosphors in the second biological transparency window via energy transfer. Inherence in this approach to material design involves an ingenious engineering for hybridizing the coordination networks of hosts, tailoring the topochemical configuration of dopants, and bridging a cascaded tunnel for transferring the persistent energy from traps, to sensitizers and then to acceptors. Another significance of this endeavour is to highlight a rational scheme for functionally important hosts and dopants, Cr/Nd co-doped Zn_1−x_Ca_x_Ga_2_O_4_ solid solutions. Such solid-solution is employed as an optimized host to take advantage of its characteristic trap site level to establish an electron reservoir and network parameters for the precipitation of activators Nd^3+^ and Cr^3+^. The results reveal that the strategy employed here has the great potential, as well as opens new opportunities for future new-wavelength, NIR phosphorescent phosphors fabrication with many potential multifunctional bio-imaging applications.

There is an increasing interest in the use of long persistent phosphorescence in the biologically transparent window to drive the photonic bioprobe for tracing the cancer cells^1^. Long phosphorescent phosphors (LPPs) can help avoiding the challenging requirement of high-intensity illumination during the signal collection, which often leads to decreased signal-to-noise ratio and photon-induced deterioration of analytes^2^. This emerging research trend, which incorporates various fields of materials science, biology, chemistry, engineering, physics and pharmaceuticals, follows two main directions: operation waveband and persistent duration, with many relevant crossing points in between^3^,^4^. As we know, there are two biologically transparent windows: first one at 650–950 nm and second one at 1000–1350 nm^5^; Near-infrared (NIR) light in the first transparency window can penetrate biological tissues such as skin and blood more efficiently than visible light^6^, yet the second region has even lower absorption and scattering therefore offers more efficient tissue penetration^7^. However, the main researches about the operational waveband of NIR LPPs mainly focus on the short wavelength region, i.e. first NIR window.

In addition to altering the emission center and tailoring the crystal field surrounding the activator, another useful strategy to extend the operational waveband, is to transfer the persistent energy of sensitizers to acceptors^8^. In fact, although the afterglow properties are predominantly controlled by the active traps, more subtle effects, such as topochemical coordination-configuration of dopant ions, can also have a profound role to the spectroscopic features of LPPs, which has long been recognized as a significant issue lying at the heart of doping chemistry and photoluminescent theory^9^. Considering the advanced engineering of cascaded tunnel of energy transfer (traps → activator(A) → activator(B)) and going into the details of it, one has at one’s disposal several decades worth of well-established principles in the coincident matching of macroscopical and microscopic features in spectroscopy, coordination chemistry and network connectivity relating to activators and hosts^10^,^11^. Traditionally, materials scientists view such network-engineering design accessed via active impurities with a practical eye intent on describing integral architectures in terms of ion types, valency and radius, local coordination geometries, as well as their concomitant implications for electronegativity and chemical bonding^12^. However, due to the complex attribute of topological network, there are still remaining grand challenges: to gain better modulation for the local coordination configuration of dopants, to understand the principle linking the indispensable transfer channel of independent individual, and to realize true predictability to the arrangements of traps and dopants (sensitizers, activators, or co-dopants) in coordinated network.

In this work we present a new “Top-down” approach to design and synthesize the long phosphorescent phosphors in the second biological transparent window. The material design approach employed here involves an ingenious engineering for hybridizing the coordination networks of hosts, tailoring the topochemical configuration of dopants, and bridging cascaded channels for transferring the persistent energy from traps, to sensitizers, to acceptors. We present a closed energy transfer channel from Cr^3+^ to Nd^3+^ in ZnGa_2_O_4_ phosphor and invalid electronic reservoir in CaGa_2_O_4_ phosphor, respectively. Persistent energy-transfer could occur in Zn_1–x_Ca_x_Ga_2_O_4_ solid-solution because two dopants were successfully locked in a cage via the efficient crystal packing at an appropriate distance, in addition to the preservation of native electron traps. The hybrid network topologies and structural motifs, thus far will be outlined with particular emphasis on how specific route of energy transfer can be prepared via premeditatedly designing a material system. Such design strategy will notably open a vista of potential avenues for the design of new optical functional materials for the future.

## Results and Discussion

**Figure 1:**
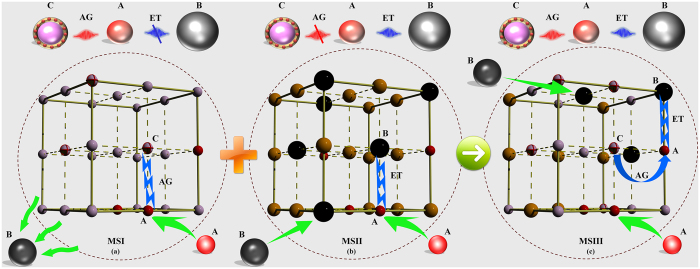
Schematic illustration showing the influence of ions doping-pattern and network-structural motifs on energy transfer process between traps and dopants. (**a,b**) represent the typical trapping and de-trapping process (AG) (traps (C) → activator (A)), as well as energy transfer process (ET) (activator (A) → activator (B)) in different material system (MSI and MSII), respectively. Hybrid Materials (MSIII) involves a solid-solution to offer the suitable coordination geometry for activators (A) and (B), and realize the cascaded energy transfer (traps (C) → activator (A) → activator (B)).

Our strategy was inspired by the fundamental spectroscopic theory of energy transfer and local intercalation reaction in inorganic polycrystals (Fig. 1)^13^. In our view, a typical long phosphorescent phosphor (MSI, in Fig. 1a) features a prominent electron reservoir (C, in Fig. 1) with the distinct ability of storing and releasing the captured electrons, as well as a notable photon-emitter (A, in Fig. 1) with higher quantum efficiency under the condition of accurately matching lattice-coordination network and atomic radius^14^. A pre-established electronic transfer channel (AG, traps (C) → activator (A), in Fig. 1) ensures the long persistent phosphorescence. However, the topological network does not provide an opportunity for another activator (B, in Fig. 1) to embed itself into the suitable lattice site. Such structural constraint thus, closes the possible channel of energy transfer (ET, in Fig. 1) between (A) and (B), leading to the luminescent and phosphorescent quenching. Fortunately, the existing chemical and spectroscopic knowledge offer a far-sighted technique to select another material system (MSII, in Fig. 1b), which allows a synchronous precipitation of activator (A) and (B), as well as engineers a theoretically existent energy-transfer channel (activator (A) → activator (B)). But to our surprise, this scheme misses the necessary electron reservoir so as to completely decrease the probability of electrons trapping-detrapping (Fig. 1b).

The use of solid-solution complexes to engineer predictable, multi-dimensional infinite networks has received ever-increasing attention in the area of chemistry and materials science^15^. Solid-solutions have already proven their superiorities in the areas of optical, optoelectronic, electrical and magnetic properties than the single component^16^. Pan *et al.* broke new ground in the field by using zinc gallogermanates solid-solution as the system, thereby achieving a super-long NIR afterglow emission time of 360 h^3^. Kobayashi *et al.* also demonstrated a state-of-the-art Li_x_FePO_4_ solid-solution technology, opening the door for lithium ion batteries to take their place in large-scale applications^17^. In addition, a series of solid-solution, such as AgGa_1−x_Al_x_O_2_, Zn_1−x_Cu_x_S, (SrTiO_3_)_1−x_(LaTiO_2_N)_x_, also have been developed and used as the advanced photocatalysts to enhance the photocatalytic activity of a given semiconductor photocatalyst^18^. Therefore, solid-solution highlights hybrid coordination network of host, and is expected to open up a possibility in the visualization of the structural and functional binding process of traps and all activators into an independent system^19^. By rationally deploying an indirect intercalation complex comprised by polyhedron ligands of materials (MSI) and (MSII), hybrid coordination-network of novel solid-solution (MSIII, in Fig. 1c) is engineered to steady the activators (A) and (B), modulate the topochemical configuration of activators, and realize the cascaded energy transfers, traps(C) → activator(A) → activator(B) (Fig. 1c). Such novel structural motif is anticipated to adopt a disturbance to native unit cell and bridging a predictable periodic coordination network.

**Figure 2:**
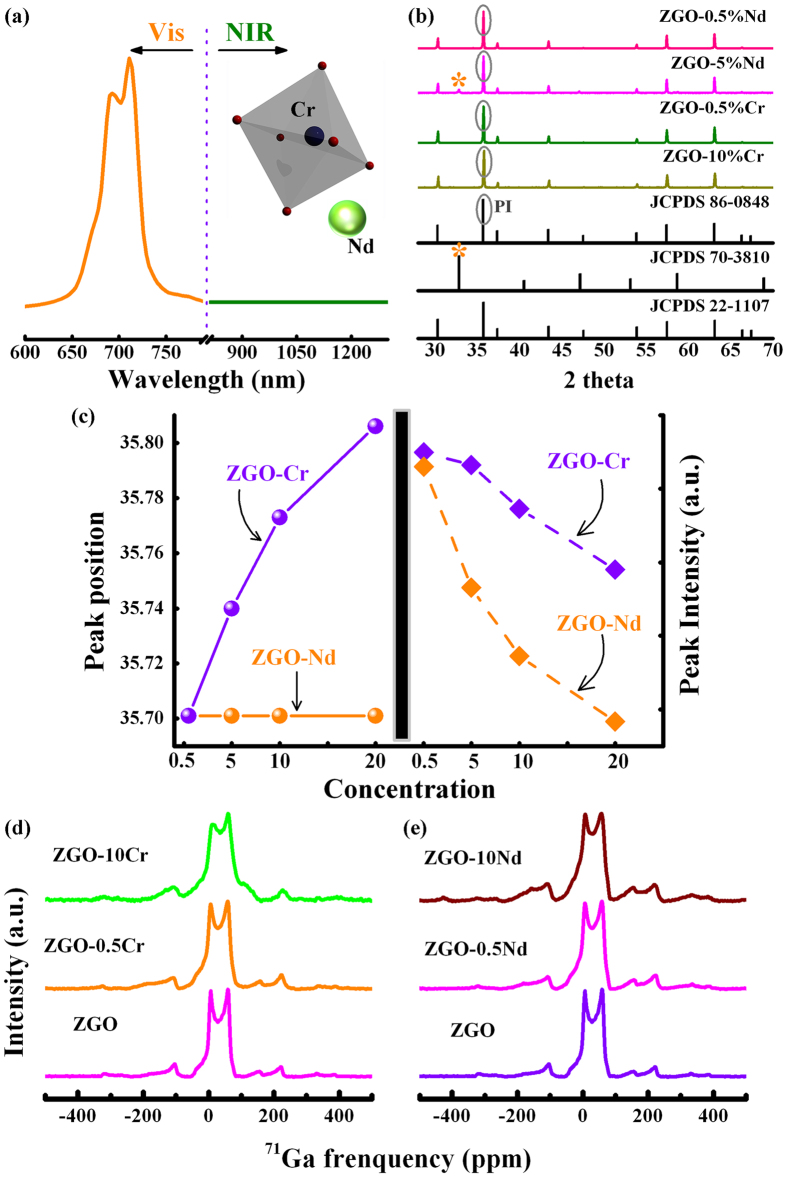
(**a**) Vis-NIR long persistent phosphorescence spectrum of ZnGa_2_O_4_: Cr/Nd phosphor. The inset shows a conjectural doping pattern of Cr and Nd. (**b**) XRD patterns for ZnGa_2_O_4_: xCr (x = 0.5% and 10%) and ZnGa_2_O_4_: xNd (x = 0.5% and 5%) phosphors. (**c**) Dependence of XRD peak ([PI] labeled in Fig. 2b) position and intensity as a function of Cr and Nd concentration in ZnGa_2_O_4_ phosphor. (**d–e**) ^71^Ga solid state NMR spectra of ZnGa_2_O_4_: xCr (x = 0.5% and 10%) and ZnGa_2_O_4_: xNd (x = 0.5% and 10%) samples. All spectra were recorded at a magnetic field of 11.7 T with a sample spinning frequency of 25 kHz.

To validate research idea, a typical NIR long phosphorescent phosphor ZnGa_2_O_4_: Cr was pursued as preferential material system, which has been proven capable of supporting high defect densities, thought to be associated primarily with Zn vacancies (V_Zn_) and O vacancies (V_O_), as well as some antisite deficiencies (Zn_Ga_)^20^. Making use of its defect capacity, ZnGa_2_O_4_: Cr has been demonstrated as a NIR photo-emitter with surprisingly long persistent phosphorescence in first NIR window (Supplementary Fig. S1). Here, we target the operating waveband in the second NIR window by transferring the persistent energy of Cr^3+^ to Nd^3+^ in Cr/Nd-codoped ZnGa_2_O_4_ LPPs. Nd^3+^ ion is chosen as the emission center in order to take advantage of the appropriate energy level characteristic, i.e. NIR-absorption (680, 750 and 800 nm) and NIR emission (1064 nm)^21^. The various sharp transitions of Nd^3+^ [^4^I_9/2_ → ^4^F_5/2_], [^4^I_9/2_ → ^4^F_7/2_], [^4^I_9/2_ → ^4^F_9/2_], just overlap the electron transition from metastable state (^4^T_2_) to ground state (^4^A_2_) of Cr^3+^, allowing the potential energy transfer from Cr to Nd^22^. However, no any NIR phosphorescence in the second NIR window can be observed in Cr/Nd-codoped ZnGa_2_O_4_ LPPs (Fig. 2a). In fact, the desired phosphorescence is still absent in Nd^3+^ singly doped ZnGa_2_O_4_ phosphor after ceasing the excitation (Supplementary Fig. S2). It is notable that the diffuse reflection spectrum consists of the characteristic transition bands centered at 530, 588, 688, 748 and 808 nm, respectively, corresponding to Nd^3+^ f-f transition, [^4^I_9/2_ → ^2^K_13/2_ + ^4^G_7/2_ + ^4^G_9/2_], [^4^I_9/2_ → ^2^G_7/2_ + ^2^G_5/2_], [^4^I_9/2_ → ^4^F_9/2_], [^4^I_9/2_ → ^4^F_7/2_], [^4^I_9/2_ → ^4^F_5/2_] in Nd^3+^ doped ZnGa_2_O_4_ phosphor (Supplementary Fig. S3)^21^; yet under the excitation at 748 nm, emission peak at 1064 nm, attributed to Nd^3+^ [^4^F_3/2_ → ^4^I_11/2_] transition is not identifiable (Supplementary Fig. S4). This attractive optical quenching-phenomenon of luminescence and phosphorescence may be not concerned with the trap distribution, but the microcosmic network architecture.

In ZnGa_2_O_4_, a majority of [Ga^VI^] cations occupy octahedral sites, whereas all of the [Zn^IV^] cations occupy tetrahedral sites^23^. As a preliminary conjecture, Cr^3+^ has proven its strong ability to substitute for Ga^3+^ in distorted octahedral coordination, whereas Nd^3+^ cannot be effectively introduced into this specific network configuration (inset of Fig. 2a). In order to identify this possibility of ion doping, we focus on the intricate topochemical coordination geometry of Cr and Nd ions in zinc gallate spinel. The elucidation is performed in detail by a combination of XRD data and ^71^Ga solid state nuclear magnetic resonance (NMR) studies. XRD patterns of ZnGa_2_O_4_: xCr (x = 0.5%, 5%, 10% and 20%) and ZnGa_2_O_4_: xNd (x = 0.5%, 5%, 10% and 20%) phosphors were measured and shown in Fig. 2b, Supplementary Fig. S5, S6. The peaks in XRD patterns of all Cr-doped samples are well indexed to pure ZnGa_2_O_4_ spinel structure (JCPDS 86-0848). In stark contrast, the higher doping content (up to 5%) of Nd ion gives rise to an impure phase, NdGaO_3_ (JCPDS, 70-3810) in Nd-doped samples. Another interesting phenomenon, i.e. XRD dominated peak (PI in Fig. 2b) shifting towards to higher 2θ value with the increment of Cr content, reveals a small linear variation in ZnGa_2_O_4 _unit cell lattice parameter with Cr^3+^ substitution, whereas no any shift of same peak is observed in Nd-doped samples, further ensuring the distinct phase splitting (Fig. 2c). In addition, a decline of the peak intensity in Fig. 2c also is present. Nevertheless, the causes of this decline may be different and rooted from either the substitution or the phase splitting.

NMR allows the observation of specific quantum mechanical and magnetic properties of atomic nucleus, as well as provides the detailed information about the structure, dynamics, reaction state, and chemical environment of molecules^24^. Many scientific techniques exploit NMR phenomena to cover the interplay between the ligands and geometric centers, as well as study the topological network motif in crystals, microcrystalline powders, or anisotropic solutions, etc^25^. ^71^Ga solid-state NMR is famous for the permission of quantitative analyses to different Ga^3+^ central coordination state in inorganic solids^26^. Figure 2d,e shows the systematical physical investigations of Ga coordination geometry in Cr and Nd singly doped ZnGa_2_O_4_, respectively. For the undoped ZnGa_2_O_4_ samples, ^71^Ga NMR spectra exhibit two well-resolved resonances. The relative higher intensive signal at about 31 ppm is characteristic of sixfold coordinated Ga atoms, and the other weaker one ~at 170 ppm corresponds to Ga atoms in the tetrahedral sites of the spinel structure^26^. It is necessary to mention that with increasing Cr content (from 0.5% to 10%), ^71^Ga NMR spectra present a significant broadening of spectral lines (Fig. 2d). In prominent contrast, scarcely any distinct influence on NMR spectral lines can be found by varying the Nd doping content in solid NMR spectra of ZnGa_2_O_4_: xNd (x = 0.5% and 10%) phosphors (Fig. 2e). The clear separation of NMR chemical shift at ~31 ppm between the two samples implies the precipitation of Cr^3+^ into the octahedral lattice site and the excludability of local configuration to Nd^3+^ ions. The NMR results are in accordance with XRD data, offering a powerful structural evidence to explain the interesting phenomena of phase splitting and luminescence quenching.

Actually, rare-earth elements generally form complexes which have high coordination numbers (CNs) and weak metal-ligand bonds, because of their large ionic radii and relatively low oxidation states^27^. Typically transition-metal and main-group elements have coordination numbers 2–6, while rare-earth metals have CNs > 6^28^. The resulting coordination polyhedra include trigonal prisms (CN = 6) or its variation by stepwise capping of the prism face up to CN = 9, in addition to square antiprisms (CN = 8); Coordination number 3 is realized only under extreme conditions^28^. Therefore, to supply an ideal dwelling for Nd^3+^, a suitable material system should be proposed. Alkaline-earth metals have large ionic radii and various coordination-numbers 3–8 in different hosts, which ensure the selection of alkaline-earth gallates^29^. CaGa_2_O_4_ has a similar spinel crystal structure with ZnGa_2_O_4_. In CaGa_2_O_4_, [Ca^VI^] cations occupy octahedral sites^29^. This configuration thus features a path of easy doping ion precipitation into the octahedral [Ca^VI^] under the condition of matching geometrical lattice and atomic radius, which occurs with rare earth ion, Nd.

**Figure 3:**
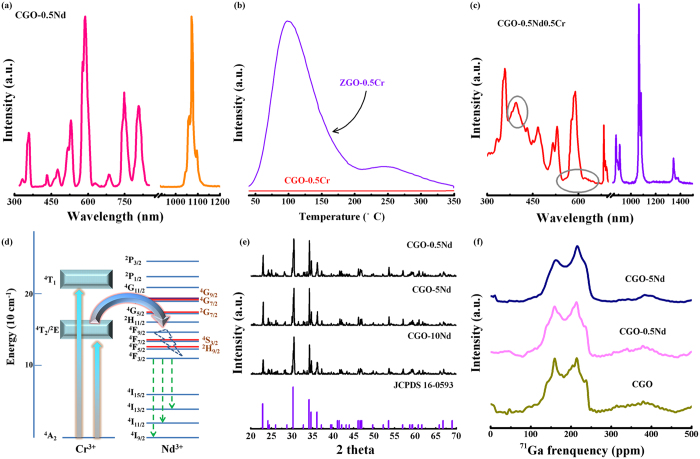
(**a**) Static photoluminescence spectrum under excitation at 750 nm and the corresponding photoluminescence excitation spectrum monitored at 1064 nm of CGO-0.5Nd phosphor; (**b**) Thermoluminescence curves of 0.5%Cr-doped ZnGa_2_O_4_ and CaGa_2_O_4_ phosphors measured 30 s after irradiation ceased; (**c**) Photoluminescence spectrum under excitation at 410 nm and photoluminescence excitation spectrum monitored at 1064 nm of CGO-0.5Cr0.5Nd phosphor; (**d**) Schematic illustration showing the energy-level diagram of Cr^3+^ and Nd^3+^ in CaGa_2_O_4_ phosphors; (**e**) XRD patterns for CaGa_2_O_4_: xNd (x = 0.5%, 5% and 10%) phosphors; (**f**) ^71^Ga NMR spectra of CaGa_2_O_4_: xNd (x = 0, 0.5% and 5%) samples.

As expected, Fig. 3a exhibits the characteristic transitions of Nd^3+^ in Nd singly doped CaGa_2_O_4_ phosphor. However, the idealistic and aspirational long persistent phosphorescence is still absence in Cr singly, Nd singly and Cr/Nd doped CaGa_2_O_4_ phosphors, respectively (Supplementary Fig. S7). A possible cause of this problem is due to the lack of effective traps (Fig. 3b). In sharp contrast to Cr/Nd codoping ZnGa_2_O_4_, photoluminescence excitation (PLE) spectrum monitored at 1064 nm of Cr/Nd codoping CaGa_2_O_4_ sample consists of two specific excitation bands centered at ~410 and ~620 nm, in addition to Nd^3+^ characteristic f-f transitions (Fig. 3c), indicating an energy transfer from Cr^3+^ to Nd^3+^. Obviously, the strong one is attributed to the Cr^3+^ [^4^A_2_ → ^4^T_1_], while the weak one corresponds to Cr^3+^ [^4^A_2_ → ^4^T_2_]^30^. Further verification of energy transfer between Cr^3+^ and Nd^3+^ is supplied by emission spectrum and decay curve monitored at 1064 nm under the excitation wavelength at 410 nm (Fig. 3c and Supplementary Fig. S8). A possible channel of energy transfer from Cr^3+^ to Nd^3+^ is Cr^3+^ [^4^T_2_ → ^4^A_2_]: Nd^3+^ [^4^I_9/2_ → ^4^F_5/2_], [^4^I_9/2_ → ^4^F_7/2_], or [^4^I_9/2_ → ^4^F_9/2_], depending on the overlap between Cr^3+^ emission band and Nd^3+^ absorption band (Fig. 3d)^31^. As discussed above, due to the similar atomic radius and geometric configurations, Nd ions can easily precipitate on Ca lattice site in CaGa_2_O_4_, enabling the distinct photoluminescence (PL). To probe the lattice configuration and substitution progress in CaGa_2_O_4_, we performed XRD and solid state NMR experiments. X-ray diffraction pattern first confirms the crystallization of Nd-doped calcium gallate (Fig. 3e). In contrast to Nd-doped ZnGa_2_O_4_, all Nd-doped CaGa_2_O_4_ samples can be indexed as standard phase CaGa_2_O_4_ (JCPDS 16-0593). There is no any apparent observation of phase splitting from XRD data, even under a higher doping content of Nd^3+^, firmly supporting the rational inclusion of Nd^3+^ into an inert matrix, CaGa_2_O_4_. This result is also supported by ^71^Ga solid state NMR spectra. In contrast to ZnGa_2_O_4_ host, the undoped CaGa_2_O_4_ sample has a dominant chemical shift at 170 ppm (Fig. 3f). With increasing dopants content, CaGa_2_O_4_: Nd also has the same effect of NMR resonances’ line broadening and the linear increase of NMR resonances integrated intensity, strongly suggesting the successful substitution in substantial amounts of Nd into Ca lattice site.

**Figure 4:**
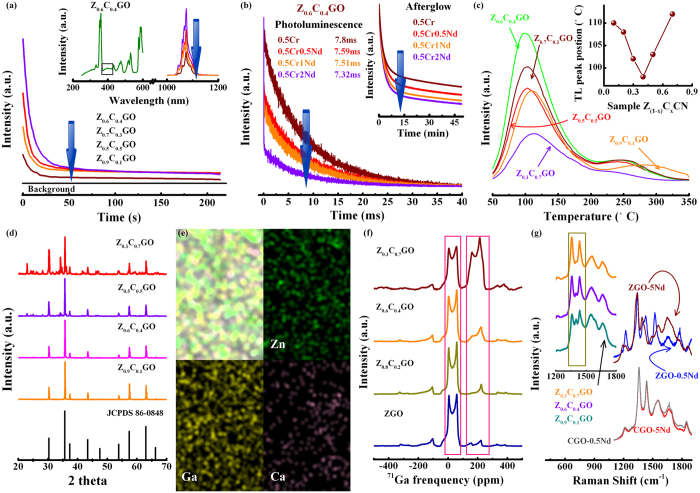
(**a**) persistence time monitored at 1064 nm as a function of Ca concentration (x = 0.1, 0.3, 0.4 and 0.5). The inset shows the long persistent phosphorescence spectra of Z_1-x_C_x_GO (x = 0.1, 0.3, 0.4 and 0.5) samples and photoluminescence excitation spectrum monitored at 1064 nm of sample Z_0.6_C_0.4_GO. (**b**) Normalized photoluminescent and phosphorescent decay curves of Z_0.6_C_0.4_GO: 0.5%Cr/xNd (x = 0, 0.5%, 1% and 2%) samples. The monitored transition is Cr^3+^ [^4^T_2_ → ^4^A_2_]. (**c**) Thermoluminescence curves of Z_1-x_C_x_GO (x = 0.1, 0.3, 0.4, 0.5 and 0.7) phosphors measured 30 s after irradiation ceased. The inset shows the dependence of TL peak position as a function of Ca concentration. (**d**) XRD patterns for Z_1-x_C_x_GO (x = 0.1, 0.4, 0.5 and 0.7) phosphors. (**e**) EDX mapping of sample Z_0.6_C_0.4_GO. (**f**) ^71^Ga NMR spectra of Z_1-x_C_x_GO (x = 0, 0.2, 0.4 and 0.7) phosphors. (**g**) Normalized Raman spectra of ZGO-0.5Nd, ZGO-5Nd, CGO-0.5Nd, CGO-5Nd and Z_1-x_C_x_GO (x = 0.1, 0.4 and 0.7) phosphors.

Seemingly, as the individual backbone, MGa_2_O_4_ (Zn and Ca) polymorph is chosen as the prototypical coordination network for its respective ability to engineer the functionally independent tunnel, traps(C) → activator(A), or activator(A) → activator(B), used to transfer the required energy. The only regret is the fundamentally missing connection of traps(C) → activator(A) → activator(B) in a separate material system. To address this issue, we anticipate a novel solid-solution Zn_1-x_Ca_x_Ga_2_O_4_ to bridge a new channel for transferring the persistent energy from traps to desired ions, based on the cautious consideration for crystal structure, ion valency and chemical bond relating to hosts and dopants. The desired NIR phosphorescence at 1064 nm is finally present in the afterglow spectra of Zn_1-x_Ca_x_Ga_2_O_4_ (x = 0.1, 0.3, 0.4 and 0.5) solid-solution (Fig. 4a). Significantly, we also observe a strong dependence (i.e. rising first followed by a decline) of phosphorescent peak intensity and decay dynamics on Ca concentration in Fig. 4a. We attribute this special spectral change of Nd^3+^ to the successful persistent energy transfer from Cr^3+^ to Nd^3+^, which is supported by the meticulous spectral studies of Nd^3+^ in an optimal Zn_0.6_Ca_0.4_Ga_2_O_4_: 0.5Cr/0.5Nd solid-solution: PLE band at 410 nm should be assigned to Cr^3+^ transition [^4^A_2_−^4^T_2_], while a distinct NIR PL peak at 1064 nm is observed under the excitation at 410 and 600 nm (Fig. 4a and Supplementary Fig. S9). The additional support for the formation of an unrestricted energy tunnel, traps → Cr^3+^ → Nd^3+^, is the analysis of kinetic processes in Z_0.6_C_0.4_GO: 0.5%Cr/xNd (x = 0, 0.5%, 1% and 2%) samples (Fig. 4b). PL decay dynamics study of Cr^3+^ shows a notable shortening in decay lifetime from 7.8 (Z_0.6_C_0.4_GO−0.5Cr), to 7.59 (Z_0.6_C_0.4_GO−0.5Cr0.5Nd), to 7.32 ms (Z_0.6_C_0.4_GO−0.5Cr2Nd), giving clear evidence of successfully simultaneous precipitation of two activators into the corresponding lattice along with the effective energy transfer from Cr^3+^ to Nd^3+^.

It should be noted that, to the best of our knowledge, this type of NIR long-persistence phosphorescence has not been previously reported to occur in hybrid coordination networks by engineering cascaded energy transfer channels. Such substantial progress is strongly influenced by two key attributes; one is trap distribution and another is network architecture. Apparently, the variation of trap distribution may be not a crucial factor in exploring the nature of transfer channel, because the indispensable electron reservoir is still steadily embedded in all the Zn_1-x_Ca_x_Ga_2_O_4_ solid-solutions (Fig. 4c). To probe the evolution of topological network-dependent topochemical coordination, the systematic characterization, such as, XRD, solid NMR, EDX mapping and Raman spectra should be conducted^32^. XRD peaks in Z_1-x_C_x_GO (x = 0.1, 0.4, 0.5 and 0.7) samples indicate their ZnGa_2_O_4_ spinel solid-solution nature, while the superimposed peaks in samples Z_0.5_C_0.5_GO and Z_0.3_C_0.7_GO can be well indexed by the diffraction peaks of ZnGa_2_O_4_ and CaGa_2_O_4_ (Fig. 4d, and Supplementary Fig. S10). EDX mapping analysis reveals the solid-solutions have uniform distribution of Ca elements in all of the spinel solid-solutions (Supplementary Fig. S11, S12 and Fig. 4e). EDX experimental composition approximating the theoretical value supports the successful inclusion of Ca elements in spinel crystals (Supplementary Table S2).

^71^Ga NMR spectra have provided some insights into the coordination variation of Ga center in ZnGa_2_O_4_ and CaGa_2_O_4_ phosphors, due to the incorporation of Cr and Nd. it is also expected to manifest its ability in resolving the question of topochemical configuration’s evolution process, as the addition of Ca element. As shown in Fig. 4f, with increasing Ca content (0, 0.2, 0.4 and 0.7), two resonances at 170 and 31 ppm in ^71^Ga NMR spectra increasingly present the linear broadening. In these solid solutions, Zn-O and Ga-O tetrahedron could suppress the intrusion of Ca element due to the mismatch of coordination configuration. In fact, to steady Ca ion, parts of Ga-O octahedron must reorient to form the new polyhedron network Ca-O octahedron along with the transformation from Ga-O octahedron to Ga-O tetrahedron due to the decrease of Zn-O tetrahedron. In detail, for the samples Z_1-x_C_x_GO (x = 0, 0.2 ,0.4), the motion of local hybrid coordination-networks evolution include: (1) the precipitation of Ca on the lattice site of octahedron Ga, giving rise to the broadening of NMR resonance at 164 ppm; (2) the conversion from Ga-O octahedron to Ga-O tetrahedron, resulting in the enhancement of NMR resonance at 65 ppm. This interesting redeployment of network configuration thus permits the modification of topochemical state of dopants, as well as opens the possibility of bridging cascaded channels to transfer the persistent energy. To further validate the research idea aiming at the network configuration, Raman spectra of the fabricated samples also can be selected as the pertinent tool to further analyze the evolution of network architecture (Fig. 4g and Supplementary Fig. S13)^33^. In stark contrast to samples ZGO-0.5Nd and ZGO-5Nd, normalized Raman spectra of samples CGO-0.5Nd and CGO-5Nd do not exhibit the notable Raman peak shift and variation of Raman peak intensity, indicating a strong constraint of topological network to the migration of Nd ions in CaGa_2_O_4_. In fact, only two distinct Raman bands at ~1358 and 1434 cm^−1^ are present in the Raman spectrum of CGO-0.5Nd, while the Raman spectrum of ZGO-0.5Nd includes three identifiable Raman peaks at ~1341, 1389 and 1425 cm^−1^. Thus, Raman spectra of Z_1-x_C_x_GO (x = 0.1, 0.4 and 0.7) solid-solutions consequentially show a unit number decrease of Raman peaks with the increment of Ca content (inset of Fig. 4g). The variation of middle peak at 1401 cm^−1^ as a function of Ca doping content ensures the strong signature of the hybrid network structure, which is in accordance with the XRD and solid-state NMR data.

In summary, we report a principle of bridging cascaded energy transfer channels to activate long persistent phosphorescence in the second biological window and fabrication of novel near-infrared phosphorescent phosphor Cr/Nd codoped Zn_1-x_Ca_x_Ga_2_O_4_ solid-solutions. Structural studies offer the powerfully fundamental evidences to explain the closed energy transfer channel from Cr^3+^ to Nd^3+^ in ZnGa_2_O_4_ phosphor and invalidation of electronic reservoir in CaGa_2_O_4_ phosphor. We believe that the ingenious solid-solution technology featuring the superiority of engineering a hybrid coordination-network opens new paths for advanced dynamic management of activation energy and gives the inspiration to design future new-wavelength, NIR phosphorescent phosphors by energy transfer.

## Methods

### Materials

4N pure CaCO_3_, Ga_2_O_3_, ZnO, Nd_2_O_3_ and Cr_2_O_3_ were selected as the raw materials.

### Preparation of ZnGa_2_O_4_: xCr/yNd

Phosphors with molar compositions of ZnGa_2_O_4_: xCr/yNd (x = 0, 0.5%, 5%, 10%, 20%; y = 0, 0.5%, 5%, 10%, 20%), (Supplementary Table S1) were prepared by the solid state reaction method. The reaction included a two-step thermal treatment (i.e., initial calcination at 800 °C for 5 h, secondary calcination at 1350 °C for 3 h).

### Preparation of CaGa_2_O_4_: xCr/yNd

Phosphors with molar compositions of CaGa_2_O_4_: xCr/yNd (x = 0, 0.5%; y = 0, 0.5%, 5%, 10%), (Supplementary Table S1) were prepared by the solid state reaction method. The reaction included a two-step thermal treatment (i.e., initial calcination at 800 °C for 5 h, secondary calcination at 1200 °C for 3 h).

### Preparation of Zn_1-x_Ca_x_Ga_2_O_4_: 0.5Cr/yNd

Phosphors with molar compositions of Zn_1-x_Ca_x_Ga_2_O_4_: 0.5Cr/yNd (y = 0, 0.5%, 1%, 2%; x = 0.1, 0.2, 0.3, 0.4, 0.5, 0.7), (Supplementary Table S1) were prepared by the solid state reaction method. The reaction included a two-step thermal treatment (i.e., initial calcination at 800 °C for 5 h, secondary calcination at 1350, 1350, 1300, 1300, 1270, 1250 °C for 3 h as a function of x, respectively).

### Characterization

The prepared materials were analyzed by X-ray diffraction (Cu/Kα) to confirm the sole crystalline phase. Room-temperature photoluminescence (PL), photoluminescence excitation (PLE) spectra, afterglow spectra and decay curves were measured with a high-resolution spectrofluorometer (UK, Edinburgh Instruments, FLS920) equipped with a 500 W Xenon lamp as an excitation source, with a Hamamatsu R928P visible photomultiplier (PMT) (250–850 nm) and a liquid nitrogen-cooled Hamamatsu R5509-72 NIR PMT as the detectors. TL glow curves and TL excitation (TLE) spectra were measured with a FJ-427A TL meter (China, Beijing) to characterize defect properties. Unless otherwise mentioned, the samples were pre-annealed at 600 K before testing, and some measurements were taken after pre-irradiating the samples for 10 min by using a xenon lamp. EDX images are characterized by a field emission scanning electron microscopy (FE-SEM), Nova NanoSEM 430. ^71^Ga Hahn echo NMR experiments were performed on Bruker Avance III spectrometers operating at magnetic fields of 111.4 T corresponding to ^71^Ga Larmor frequencies of 152.54 MHz) using Bruker 2.5 mm triple and double resonance probe heads. The 90^0 ^degree pulse length is 1.25 μm with a recycle delay of 8s. ^71^Ga chemical shifts were referenced relative to a 1.0 M aqueous solution of Ga(NO_3_)_3_. All ^71^Ga spectra were fitted using the Dmfit software. Raman spectra were collected with a Renishaw inVia Raman microscope irradiated by a visible laser at 532 nm.

## Additional Information

**How to cite this article**: Qin, X. *et al.* Hybrid coordination-network-engineering for bridging cascaded channels to activate long persistent phosphorescence in the second biological window. *Sci. Rep.* **6**, 20275; doi: 10.1038/srep20275 (2016).

## Supplementary Material

Supplementary Information
