# Supplementary material for: Hybrid coordination-network-engineering for bridging cascaded channels to activate long persistent phosphorescence in the second biological window
Source: Sci Rep. 2016 Feb 4;6:20275. doi: 10.1038/srep20275 (PMC4740745; doi:10.1038/srep20275)
Supplement: Supplementary Information [file srep20275-s1.pdf]

Hybrid coordination-network-engineering for bridging cascaded channels to activate long persistent phosphorescence in the second biological window

Xixi Qin[a], Yang Li[a,b,&,*], Ruili Zhang[c], Jinjun Ren[c], Mindaugas Gecevicius[a], Yiling Wu[a],Kaniyarakkal Sharafudeen[d],Guoping Dong[a], Shifeng Zhou[a], Zhijun Ma[a], and Jianrong Qiu[a,*]

[a]*State Key Laboratory of Luminescent Materials and Devices, Guangdong Provincial Key Laboratory of Fiber Laser Materials and Applied Techniques, South China University of Technology, Guangzhou 510640, China*

[b]*School of Chemistry and Chemical Engineering, South China University of Technology, Guangzhou 510640, China*

[c]*Shanghai institute of optics and fine mechanics, Chinese Academy of Sciences 201800, China*

[d]*Escola de Engenharia de Sao Carlos, Universidade de Sao Paulo, 13566-590, Sao Carlos, SP, Brazil*

[&]Equal contribution to this work

*Corresponding authors: qjr@scut.edu.cn;msliyang@scut.edu.cn;

Tel: +86-20-87113646

Fax: +86-20-87114204

Figures

Figure S1. Uv-vis-NIR long persistent phosphorescence spectrum of ZGO-0.5Cr.

Figure S2. Uv-vis-NIR long persistent phosphorescence spectrum of ZGO-0.5Nd.

Figure S3. Diffuse reflectance spectra of ZGO-0.5Cr, ZGO-0.5Nd and ZGO-0.5Cr0.5Nd.

Figure S4. Photoluminescence spectra excited at 360 nm of samples:ZGO-0.5Cr, and ZGO-0.5Cr0.5Nd samples. Photoluminescence spectra excited at 748 nm of samples ZGO-0.5Nd and ZGO-0.5Cr0.5Nd.

Figure S5. XRD patterns of the samples ZGO-0.5Cr, ZGO-5Cr, ZGO-10Cr and ZGO-20Cr.

Figure S6. XRD patterns of the samples ZGO-0.5Nd, ZGO-5Nd, ZGO-10Nd and ZGO-20Nd.

Figure S7. Uv-vis-NIR long persistent phosphorescence spectra of CGO-0.5Cr and CGO-0.5Nd.

Figure S8. Photoluminescence decay curve monitored at 1064 nm under the excitation wavelength of 410 nm of CGO-0.5Cr0.5Nd.

Figure S9. Photoluminescence spectrum of $Z_{0.6}C_{0.4}$GO-0.5Cr0.5Nd under excitation at 600 nm.

Figure S10. XRD patterns of $Z_{1-x}C_{x}$GO (x=0.1, 0.4, 0.5 and 0.7) phosphors.

Figure S11. (a)SEM images and (b)EDS analysis of $Z_{0.9}C_{0.1}$GO.

Figure S12. (a)SEM images and (b)EDS analysis of $Z_{0.6}C_{0.4}$GO.

Figure S13. Raman spectra of the samples (a)Nd_2O_3,(b)Ga_2O_3,(c)ZnO and (d)Cr_2O_3.

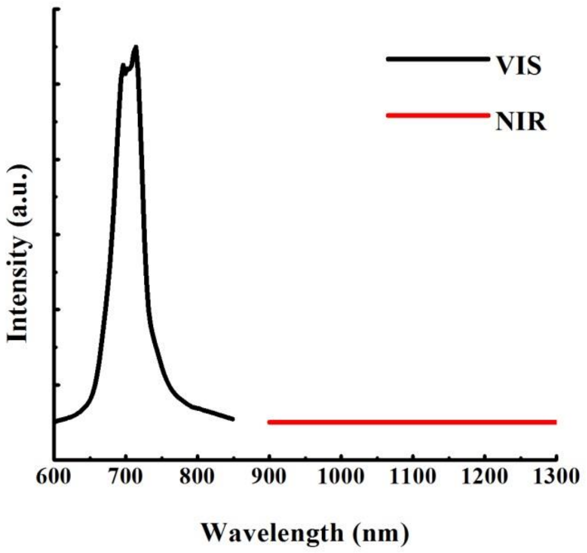
VIS
NIR
Intensity (a.u.)
600
700
800
900
1000
1100
1200
1300
Wavelength (nm)

FigureS1

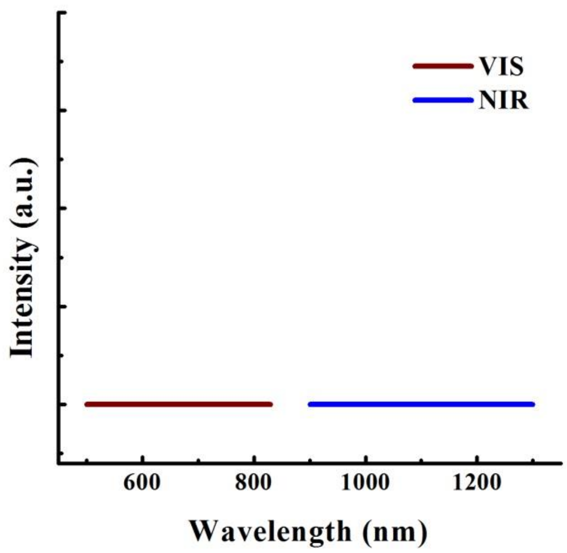

Figure S2

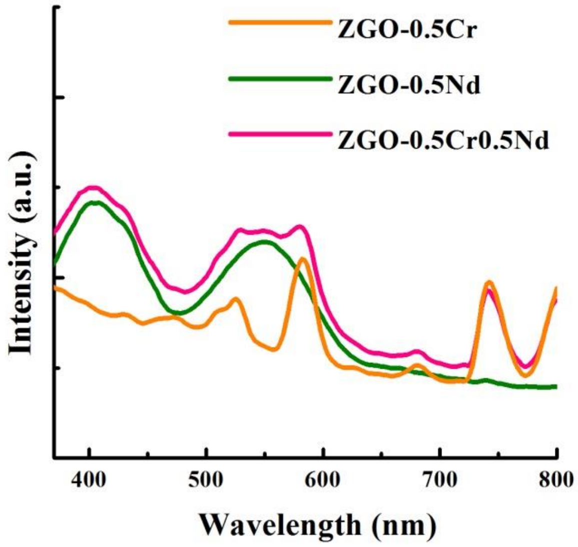
ZGO-0.5Cr
ZGO-0.5Nd
ZGO-0.5Cr0.5Nd
Intensity (a.u.)
400
500
600
700
800
Wavelength (nm)

Figure S3

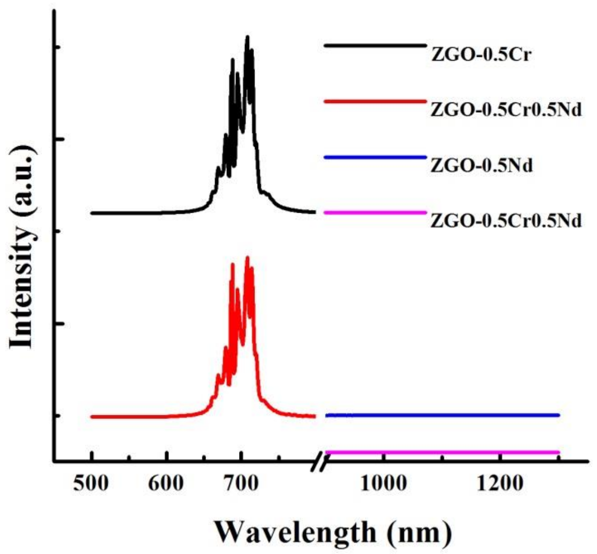
ZGO-0.5Cr
ZGO-0.5Cr0.5Nd
ZGO-0.5Nd
ZGO-0.5Cr0.5Nd
Intensity (a.u.)
500
600
700
1000
1200
Wavelength (nm)

FigureS4

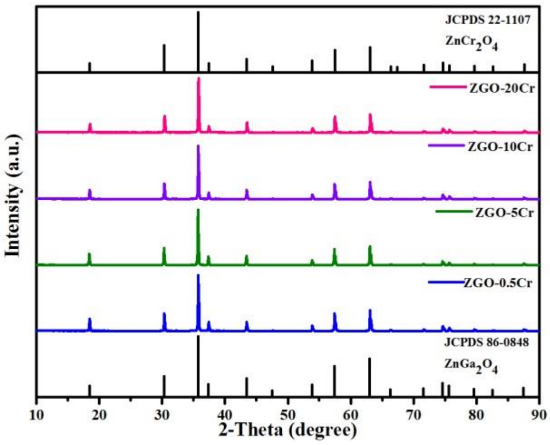
JCPDS 22-1107
ZnCr2O4
ZGO-20Cr
ZGO-10Cr
ZGO-5Cr
ZGO-0.5Cr
JCPDS 86-0848
ZnGa2O4
Intensity (a.u.)
2-Theta (degree)
10
20
30
40
50
60
70
80
90

FigureS5

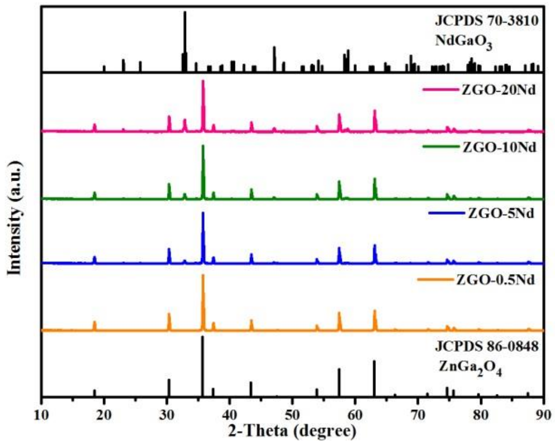
JCPDS 70-3810
NdGaO3
ZGO-20Nd
ZGO-10Nd
ZGO-5Nd
ZGO-0.5Nd
JCPDS 86-0848
ZnGa2O4
Intensity (a.u.)
2-Theta (degree)
10
20
30
40
50
60
70
80
90

FigureS6

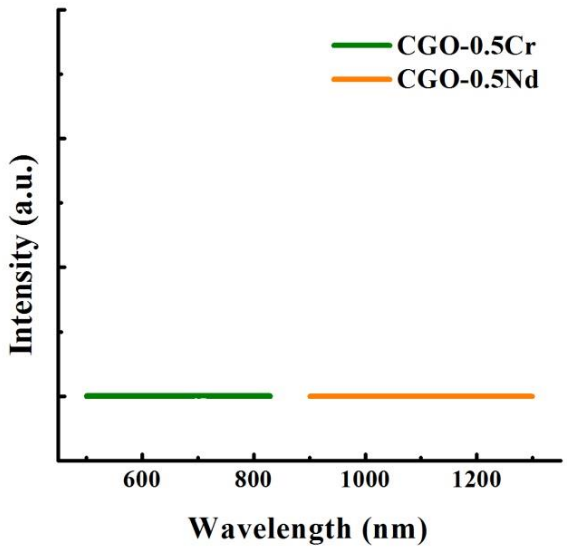
CGO-0.5Cr
CGO-0.5Nd
Intensity (a.u.)
600
800
1000
1200
Wavelength (nm)

FigureS7

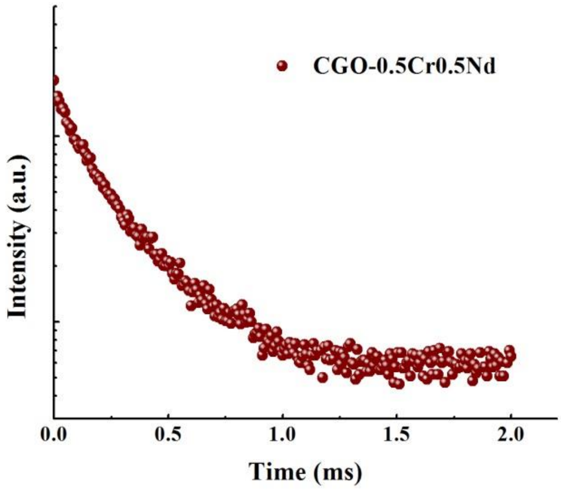

Figure S8

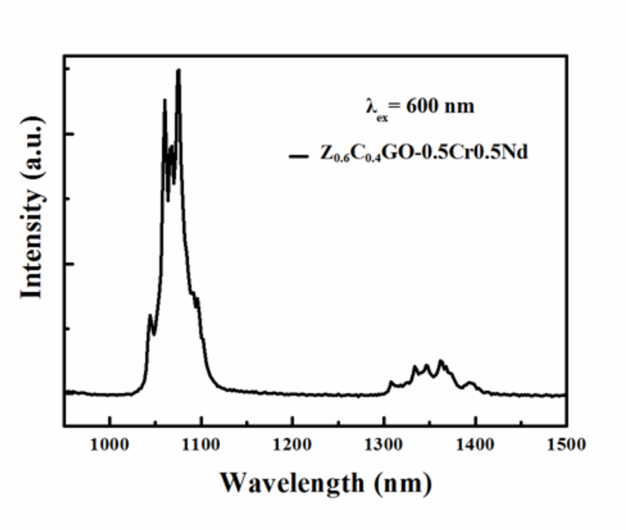

λ_{ex} = 600 nm
$Z_{0.6}C_{0.4}$GO-0.5Cr0.5Nd
Intensity (a.u.)
1000
1100
1200
1300
1400
1500
Wavelength (nm)

Figure S9

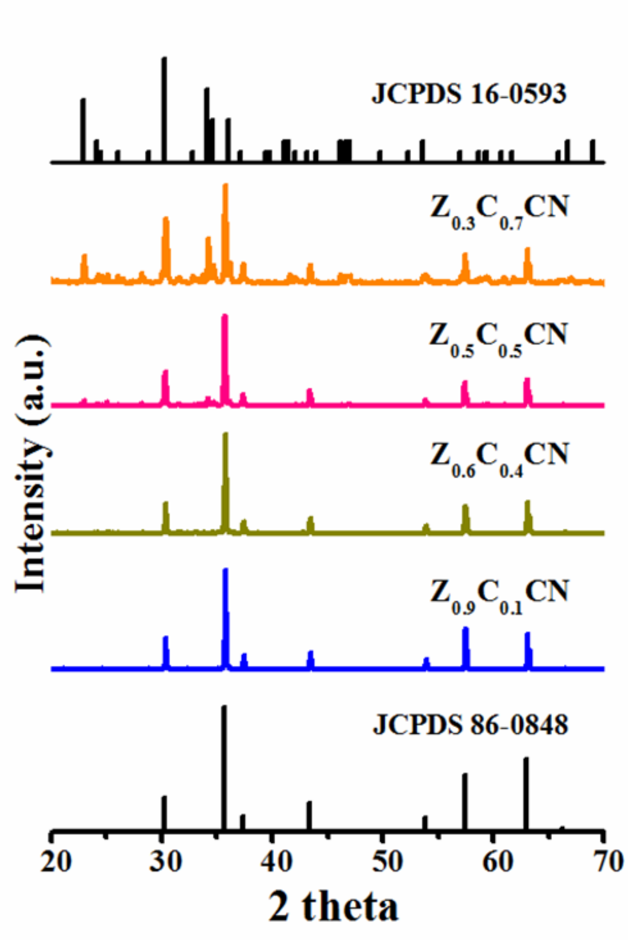
JCPDS 16-0593
Z0.3C0.7CN
Z0.5C0.5CN
Z0.6C0.4CN
Z0.9C0.1CN
JCPDS 86-0848
Intensity (a.u.)
20
30
40
50
60
70
2 theta

Figure S10

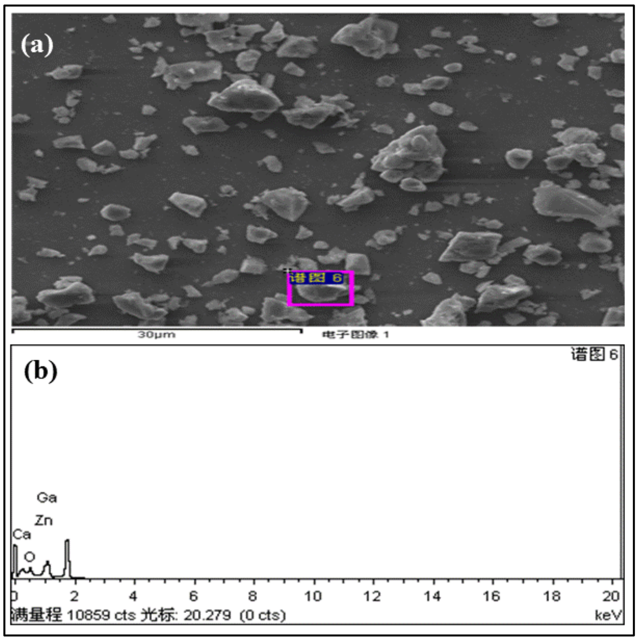
(a)
谱图 6
30µm
电子图像 1
(b)
谱图 6
Ga
Zn
Ca
O
0 2 4 6 8 10 12 14 16 18 20
满量程 10859 cts 光标: 20.279 (0 cts)
keV

FigureS11

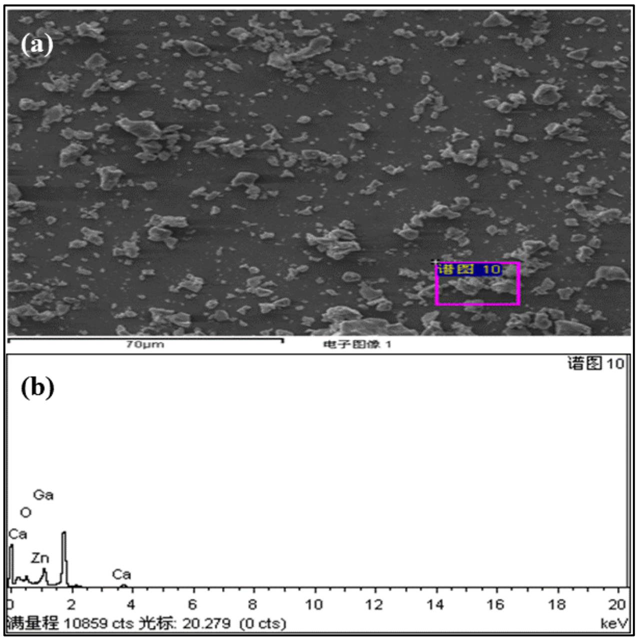
(a)
谱图 10
70µm
电子图像 1
(b)
谱图 10
Ga
O
Ca
Zn
Ca
0 2 4 6 8 10 12 14 16 18 20
满量程 10859 cts 光标: 20.279 (0 cts)
keV

FigureS12

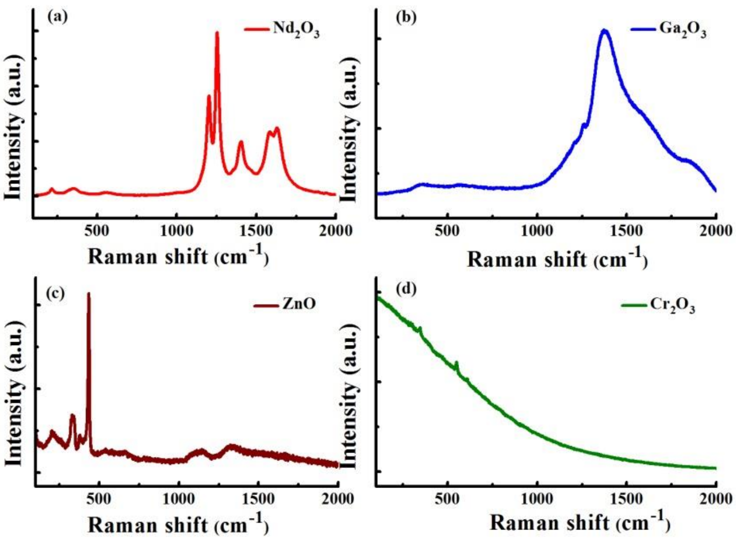
(a)
Nd2O3
Intensity (a.u.)
500
1000
1500
2000
Raman shift (cm-1)
(b)
Ga2O3
Intensity (a.u.)
500
1000
1500
2000
Raman shift (cm-1)
(c)
ZnO
Intensity (a.u.)
500
1000
1500
2000
Raman shift (cm-1)
(d)
Cr2O3
Intensity (a.u.)
500
1000
1500
2000
Raman shift (cm-1)

FigureS13

Tables

Table S1 Chemical compositions of MGa_2O_4: xCr^{3+}, yNd^{3+}(M=Ca, Zn; x=0, 0.5%, 5%, 10%, 20%; y=0, 0.5%, 5%, 10%, 20%) and $Zn_{1-x}Ca_xGa_2O_4$: 0.5%Cr^{3+}0.5%Nd^{3+}(x=0.1, 0.3, 0.4, 0.5, 0.7) phosphors.

Table S2 The comparison between theoretical and experimental composition calculated by EDS analysis of samples $Z_{0.9}C_{0.1}GO$,$Z_{0.8}C_{0.2}GO$, $Z_{0.7}C_{0.3}GO$ and $Z_{0.6}C_{0.4}GO$.

TableS1 Chemical compositions of MGa_2O_4: xCr^{3+}, yNd^{3+}(M=Ca, Zn; x=0, 0.5%, 5%, 10%, 20%; y=0, 0.5%, 5%, 10%, 20%) and $Zn_{1-x}Ca_xGa_2O_4$: 0.5%Cr^{3+}0.5%Nd^{3+}(x=0.1, 0.3, 0.4, 0.5, 0.7) phosphors.

No	Composition	No	Composition
ZGO	$ZnGa_2O_4$	CGO-5Nd	$CaGa_2O_4$: 5%Nd^{3+}
ZGO-0.5Cr	$ZnGa_2O_4$: 0.5%Cr^{3+}	CGO-10Nd	$CaGa_2O_4$: 10%Nd^{3+}
ZGO-5Cr	$ZnGa_2O_4$: 5%Cr^{3+}	CGO-0.5Cr0.5Nd	$CaGa_2O_4$: 0.5%Cr^{3+},0.5%Nd^{3+}
ZGO-10Cr	$ZnGa_2O_4$: 10%Cr^{3+}	$Z_{0.9}C_{0.1}GO$	$Zn_{0.9}Ca_{0.1}Ga_2O_4$: 0.5%Cr^{3+},0.5%Nd^{3+}
ZGO-20Cr	$ZnGa_2O_4$: 20%Cr^{3+}	$Z_{0.8}C_{0.2}GO$	$Zn_{0.8}Ca_{0.2}Ga_2O_4$: 0.5%Cr^{3+},0.5%Nd^{3+}
ZGO-0.5Nd	$ZnGa_2O_4$: 0.5%Nd^{3+}	$Z_{0.7}C_{0.3}GO$	$Zn_{0.7}Ca_{0.3}Ga_2O_4$: 0.5%Cr^{3+},0.5%Nd^{3+}
ZGO-5Nd	$ZnGa_2O_4$: 5%Nd^{3+}	$Z_{0.6}C_{0.4}GO$	$Zn_{0.6}Ca_{0.4}Ga_2O_4$: 0.5%Cr^{3+},0.5%Nd^{3+}
ZGO-10Nd	$ZnGa_2O_4$: 10%Nd^{3+}	$Z_{0.5}C_{0.5}GO$	$Zn_{0.5}Ca_{0.5}Ga_2O_4$: 0.5%Cr^{3+},0.5%Nd^{3+}
ZGO-20Nd	$ZnGa_2O_4$: 20%Nd^{3+}	$Z_{0.3}C_{0.7}GO$	$Zn_{0.3}Ca_{0.7}Ga_2O_4$: 0.5%Cr^{3+},0.5%Nd^{3+}
ZGO-0.5Cr0.5Nd	$ZnGa_2O_4$: 0.5%Cr^{3+},0.5%Nd^{3+}	$Z_{0.6}C_{0.4}GO$-0.5Cr	$Zn_{0.6}Ca_{0.4}Ga_2O_4$: 0.5%Cr^{3+},0.5%Nd^{3+}
CGO	$CaGa_2O_4$	$Z_{0.6}C_{0.4}GO$-0.5Cr 0.5Nd	$Zn_{0.6}Ca_{0.4}Ga_2O_4$: 0.5%Cr^{3+},0.5%Nd^{3+}
CGO-0.5Cr	$CaGa_2O_4$: 0.5%Cr^{3+}	$Z_{0.6}C_{0.4}GO$-0.5Cr 1Nd	$Zn_{0.6}Ca_{0.4}Ga_2O_4$: 0.5%Cr^{3+},0.5%Nd^{3+}
CGO-0.5Nd	$CaGa_2O_4$: 0.5%Nd^{3+}	$Z_{0.6}C_{0.4}GO$-0.5Cr 2Nd	$Zn_{0.6}Ca_{0.4}Ga_2O_4$: 0.5%Cr^{3+},0.5%Nd^{3+}

Table S2The comparison between theoreticaland experimentalcomposition caculated by EDS ofsamples $Z_{0.9}C_{0.1}GO$,$Z_{0.8}C_{0.2}GO$, $Z_{0.7}C_{0.3}GO$ and $Z_{0.6}C_{0.4}GO$.

Theoreticalcomposition	(%)				Experimental composition
	O	Ca	Zn	Ga	
$Zn_{0.9}Ca_{0.1}Ga_2O_4$	22.9	1.55	21.47	54.09	$Zn_{0.923}Ca_{0.108}Ga_{2.159}O_4$
$Zn_{0.8}Ca_{0.2}Ga_2O_4$	23.73	3.14	19.07	54.06	$Zn_{0.791}Ca_{0.211}Ga_{2.082}O_4$
$Zn_{0.7}Ca_{0.3}Ga_2O_4$	24.33	5.95	15.15	54.57	$Zn_{0.613}Ca_{0.391}Ga_{2.052}O_4$
$Zn_{0.6}Ca_{0.4}Ga_2O_4$	20.66	7.46	17.34	54.54	$Zn_{0.826}Ca_{0.578}Ga_{2.413}O_4$
